# Mechanical Loading Differentially Affects Osteocytes in Fibulae from Lactating Mice Compared to Osteocytes in Virgin Mice: Possible Role for Lacuna Size

**DOI:** 10.1007/s00223-018-0463-8

**Published:** 2018-08-14

**Authors:** Haniyeh Hemmatian, Rozita Jalali, Cornelis M. Semeins, Jolanda M. A. Hogervorst, G. Harry van Lenthe, Jenneke Klein-Nulend, Astrid D. Bakker

**Affiliations:** 1 Biomechanics Section, Department of Mechanical Engineering, KU Leuven, Leuven, Belgium; 2 Department of Oral Cell Biology, Academic Centre for Dentistry Amsterdam (ACTA), University of Amsterdam and Vrije Universiteit Amsterdam, Amsterdam Movement Sciences, Gustav Mahlerlaan 3004, 1081 LA Amsterdam, The Netherlands

**Keywords:** Osteocyte, Lacuna morphology, Mechanotransduction, Ex vivo mechanical loading

## Abstract

Hormonal changes during lactation are associated with profound changes in bone cell biology, such as osteocytic osteolysis, resulting in larger lacunae. Larger lacuna shape theoretically enhances the transmission of mechanical signals to osteocytes. We aimed to provide experimental evidence supporting this theory by comparing the mechanoresponse of osteocytes in the bone of lactating mice, which have enlarged lacunae due to osteocytic osteolysis, with the response of osteocytes in bone from age-matched virgin mice. The osteocyte mechanoresponse was measured in excised fibulae that were cultured in hormone-free medium for 24 h and cyclically loaded for 10 min (sinusoidal compressive load, 3000 µε, 5 Hz) by quantifying loading-related changes in *Sost* mRNA expression (qPCR) and sclerostin and β-catenin protein expression (immunohistochemistry). Loading decreased *Sost* expression by ~ threefold in fibulae of lactating mice. The loading-induced decrease in sclerostin protein expression by osteocytes was larger in lactating mice (55% decrease ± 14 (± SD), *n* = 8) than virgin mice (33% decrease ± 15, *n* = 7). Mechanical loading upregulated β-catenin expression in osteocytes in lactating mice by 3.5-fold (± 0.2, *n* = 6) which is significantly (*p* < 0.01) higher than the 1.6-fold increase in β-catenin expression by osteocytes in fibulae from virgin mice (± 0.12, *n* = 4). These results suggest that osteocytes in fibulae from lactating mice with large lacunae may respond stronger to mechanical loading than those from virgin mice. This could indicate that osteocytes residing in larger lacuna show a stronger response to mechanical loading.

**Electronic supplementary material:**

The online version of this article (10.1007/s00223-018-0463-8) contains supplementary material, which is available to authorized users.

## Introduction

Lactation is associated with large shifts in hormonal status. PTHrP and prolactin go up, PTH and estrogen go down [1]. These hormonal changes have profound effects on osteoblasts, osteoclasts, and osteocyte activity. Bone remodeling is controlled by osteocytes in response to chemical, but for an important part mechanical, stimuli. Osteocytes sense mechanical signals placed upon bone (mechanosensation), transduce the mechanical signal into a chemical response (mechanotransduction), and consequently orchestrate the activity and recruitment of osteoblasts and/or osteoclasts by producing a multitude of signaling molecules [2, 3]. It is likely that hormonal changes during lactation affect osteocyte response to mechanical signals as evidence exists that estrogen receptor-α (ERα), which is regulated by estrogen, contributes to the response of bone cells to mechanical strain [4–6] and the PTH type 1 receptor has been suggested to be an important component of mechanical signal transduction in osteocytic MLO-Y4 cells [7]. The sharp increase in PTHrP leads to a rapid expansion of the osteocyte lacunae, through a process called osteocytic osteolysis.

Osteocytes reside in cavities within the bone matrix called lacunae and interconnect through cell extensions (50–60 per cell) that reside in small canals named canaliculi [2]. Through this extensive communication network, the osteocytes sense mechanical loads upon bone. Bone matrix deformations due to mechanical loading drive an interstitial fluid flow through the canaliculi. It has been suggested that the fluid flow activates osteocytes by generating streaming potentials [8, 9], shear stress on the cell membrane [10, 11], or by creating tension along the elements attaching the cell process to the lacunar wall [12]. In addition, the inhomogeneities in the bone microstructure due to the osteocyte lacunar network can, in theory, locally amplify the matrix strain to a magnitude that is sufficient to directly activate the osteocyte cell bodies [13], thereby eliciting a biological response [3]. Building further upon these theories, the ability for osteocytes to sense mechanical stimuli placed on bone would be affected by canalicular morphology and by lacunar shape, since the latter could affect the strain amplification around the cell body [14, 15]. For example, osteocytes residing in larger lacunae could experience higher strains. As a result, osteocytes would experience a locally modified mechanical environment, possibly resulting in an altered adaptive response to mechanical loading [16]. There are data to support this hypothesis: It has been shown that the shape of osteocytes and their lacuna differs between several bone diseases; e.g., their shapes are significantly different in the tibia of individuals with osteopetrosis, osteopenia, and osteoarthritis [17]. Furthermore, evidence suggests that the osteocyte lacunae are becoming smaller and more spherical with aging [18, 19], while it has been shown that aging is associated with a reduced mechanoresponse [20–22], which suggest that altered lacunar shape is a possible cause of impaired loading-induced bone remodeling process in the elderly. Yet, up to this date, experimental evidence supporting, or refuting, the presupposition that changes in lacunar morphology as seen in lactation, aging, and disease, can underlie an altered bone responsiveness to mechanical loads is non-existent.

The aim of this study was to quantify the response of osteocytes in mechanically loaded fibulae from lactating and virgin mice of similar age, gender, and genetic background, having different lacunar morphology. Because lactation is associated with altered hormonal status, we mechanically loaded excised fibulae rather than bones in vivo, in order to equalize hormonal conditions around osteocytes for at least 24 h before applying the mechanical stimulus. We hypothesized that osteocytes in bones from lactating mice show a stronger response to the mechanical loading, measured as higher loading-related changes in sclerostin expression as well as higher protein expression of β-catenin in comparison to osteocytes from virgin mice. We chose to measure β-catenin and sclerostin as parameters for the response to loading because these are well established to alter in expression in osteocytes after loading of bone [23–25]. Considering the crucial role of osteocyte to maintain healthy bone, a better understanding of the way osteocyte shape is related to its capability to direct bone formation and resorption may help to unravel the origins of reduced bone adaptive response as seen with, for example, lactation, disease, and aging.

## Materials and Method

### Specimens

The right and left fibulae were extracted from eight 3- to 5-month-old female lactating C57BL/6 mice. Lactating mice were euthanized after lactation period. Due to osteocytic osteolysis [26, 27], these mice have enlarged lacunae as compared to age-matched virgin C57BL/6 mice, which were used as control (*N* = 8). Mice were bred at UPC, VU University Amsterdam. All mice were group housed in conventional conditions: 12-h light, 12-h dark cycle, standard diet (1% calcium, 0.76% phosphate), and water ad libitum in standard cages. Animals were kept in accordance with the regulations of the Animal Welfare committee of the VU University Amsterdam (ACTA14-02). Mice were sacrificed with a peritoneal injection of sodium pentobarbital (0.1 ml Euthesate, Ceva Sante Animale, Naaldwijk, The Netherlands).

### Nano-computed Tomography for Quantitation of Lacunar Morphology

To quantify the morphology of the osteocyte lacunae, fibulae from lactating and virgin mice (*N* = 4 per group) were fixed in 2% PBS-buffered paraformaldehyde at 4 °C. Subsequently, 1 mm of the distal part of each fibula, starting at the distal tibiofibular junction (TFJ) was scanned at a nominal resolution of 0.8 µm using a Phoenix NanoTom m (GE Sensing and Inspection Technologies, GmbH, Wunstorf, Germany) with a 180 kV/20W high-performance nanofocus X-ray tube. The samples were rotated over 360° and the X-ray settings were standardized to 75 kV and 340 µA with an exposure time of 1000 ms. A 0.2-mm aluminum filter was used to reduce beam hardening artifacts. Applying a frame averaging of 3 and image skip of 1 resulted in a total scan time of 1 h 34 min per sample. One scan produced 1300 contiguous slices with a nominal resolution of 0.8 µm. Each slice contained 1600 × 2400 pixels. The X-ray projections were reconstructed using Datos|x software to create cross-sectional images. A smoothing filter with a Gaussian window kernel (3 pixels) was used to optimize the images.

Image processing and segmentation of osteocyte lacunar network were performed using our previously reported technique [28]. Briefly, a histogram-based global thresholding method was applied to the reconstructed images to segment the mineralized tissue and nonmineralized structures. Intracortical porosity comprising of lacunae and vascular canals was segmented by inverting the image and using the 3D despeckled filter in CTAn (v.1.16.4.1, SkyScan). The objects less than 100 µm^3^ were considered to be noise, elements with a volume in the range between 100 and 2000 µm^3^ were assumed to be osteocyte lacunae, and the objects greater than 2000 µm^3^ were considered to be vascular canals. These volume limits were used in previous synchrotron-based studies [18, 29, 30] and were based on confocal microscopy measurements indicating a size between 28 and 1713 µm^3^ for each osteocyte [31]. Note that canaliculi were not segmented since their size was below the scan resolution used in this study. After segmentation, the osteocyte lacunar network was determined by quantifying individual lacuna volume (Lc.V), lacuna orientation (Lc.θ), and lacuna sphericity (Lc.Sph) using CTAn. The lacuna sphericity was calculated as the ratio of the surface area of a sphere with the same volume as the given lacuna to the lacuna surface area. The lacuna orientation was calculated relative to the fibula longitudinal axis.

### Ex Vivo Mechanical Loading Experiment

**Fig. 1:**
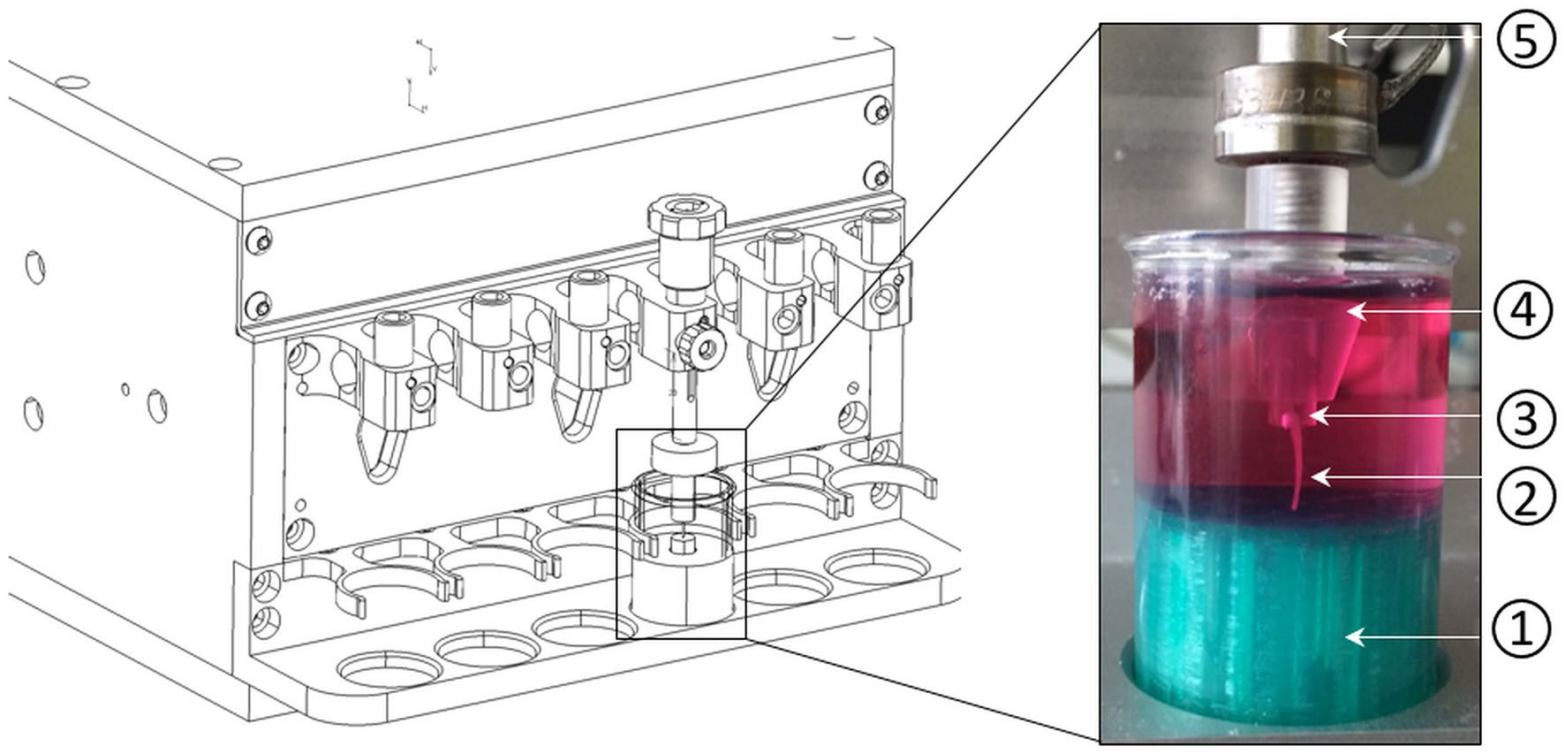
Schematic representation of the custom-made fibular loading apparatus. Main components of the fibular loading device are (1) chamber filled with Dulbecco’s modified Eagle’s medium, (2) fibula glued to a bone holder, (3) custom-made bone holder, (4) holder, and (5) microactuator

Freshly dissected fibulae (*N* = 8/group) were stripped of soft tissue. The proximal part was cut transversely and glued in a custom-made bone holder (Fig. 1) using cyanoacrylate instant adhesive (Permabond, St. Pottstown, PA, USA). The distal part of the fibulae was cut to provide a flat surface that ensured stability during loading process. Before bonding the bones to the holders, all bone holders were sterilized in 70% Ethanol for 10 min and allowed to air dry. The length of each fibula was measured using a calibrated microscope. The samples were then incubated in Dulbecco’s modified Eagle’s medium (DMEM; Gibco, Paisley, UK) supplemented with 100 U/ml penicillin (Sigma), 0.05 mg/ml streptomycin sulfate (Sigma), 1.25 µg/ml fungizone (Gibco), 5 µg/ml Vitamin C (Sigma), and 0.2% bovine serum albumin (BSA) for 24 h before loading to equalize hormone levels.

Mechanical loading of the fibulae was applied using a custom-made apparatus [32, 33] (Fig. 1), which enables to generate sinusoidal displacement with an accuracy of 0.2 µm at a rate of maximally 30 Hz using a computer-driven voice coil linear microactuator (type NCM04-25-250-2LVE; H2W Technologies, Valencia, CA). The custom-made bone holder, into which the fibula was glued, was inserted in a clamp attached to the microactuator of the loading machine and then lowered into a chamber containing culture medium as described above. The bottom of the chamber was designed to prevent any horizontal movement of the sample during loading (Fig. 1; culture chamber; green part). A pre-load of 0.01 N was applied prior to compression loading to maintain a proper contact between the fibula and the underlying surface of the chamber. Thereafter, samples were subjected to a 10-min (displacement driven) mechanical compression of 3000 µ strains at 5 Hz frequency. The strain level corresponds to peak physiological tissue strain [34], which has been shown to be anabolic for cortical bone in female mice [21]. A loading frequency of 5 Hz was chosen because the cortical bone adaptation to the mechanical loading is most efficient at loading frequency of 5–10 Hz [35]. The force–displacement data were collected using a custom-made algorithm (LabVIEW 8.2, National Instruments, Austin TX). The non-loaded fibulae were also held in place by a pre-load of 0.01 N for 10 min but without any further mechanical loading. The device was displacement driven, i.e., the applied sinusoidal displacement for each sample was determined with respect to the initial length of each fibula to achieve an equal bulk strain of 3000 µstrain for all specimens. The results of validation studies of the loading apparatus can be found as Online Resource 1.

### Quantitative PCR Analysis

Total RNA, from the 5-µm-thick longitudinal paraffin sections, was isolated using an RNeasy FFPE Kit from Qiagen (Cat.No. 73,504). Total RNA concentrations were determined with a Synergy HT (BioTek Instruments, Inc. Winooski, Vermont USA). cDNA synthesis was performed in a thermocycler GeneAmp PCR System 9700 PE (Applied Biosystems, Foster City, CA), using SuperScript™ VILO™ cDNA Synthesis Kit (Invitrogen), with 10 µl total RNA in 20 µl reaction mixture. cDNA was stored at − 20 °C until real-time PCR analysis. Real-time PCR reactions were performed using 4.0 µl cDNA and SYBR® Green Supermix (Roche Laboratories, Indianapolis, IN) in a Light Cycler® 480 (Roche Diagnostics, Basel, Switzerland). In each PCR run, the reaction mixture without cDNA was used as a negative control. For quantitative real-time PCR, the values of relative target gene expression were normalized relative to housekeeping gene 18 s and B2M expression and expressed as fold difference of loaded relative to unloaded groups. Real-time PCR was used to assess the expression of sclerostin gene (*Sost)*. All primers used for real-time PCR were from Life Technologies. Primer sequences were as follows: B2M (annealing temp 63 °C; two-step PCR reaction; 45 cycles), forward: 5′-TGCTATCCAGAAAACCCCTCAA-3′, reverse: 5′-GCGGGTGGAACTGTGTTACG-3′; 18S (annealing temp 57 °C; four-step PCR reaction; 45 cycles), forward: 5′-GTAACCCGTTGAACCCCATT-3′, reverse: 5′-CCATCCAATCGGTAGTAGCG-3′; SOST (annealing temp 56 °C; four-step PCR reaction; 45 cycles), forward: 5′-CCGTGCCTCATCTGCCTACTTG-3′, reverse: 5′-TGGCGTCATAGGGATGGTG-3′.

### Immunohistochemistry

Following mechanical loading, the fibulae were incubated overnight in culture medium. Samples were washed two times in Phosphate Buffered Saline (PBS). Then, they were fixed in 2% formaldehyde in PBS for 24 h at 4 °C. After fixation, the bone holders were removed from the fibulae. Next, the fixed bones were washed in PBS and transferred to 4% ethylenediaminetetraacetic acid (EDTA) solution in PBS at 4 °C for 2 weeks in order to decalcify. Decalcified samples were washed in PBS and sequentially dehydrated and embedded in paraffin. Thereafter, 5-µm-thick longitudinal sections were obtained. The sections were deparaffinized and incubated in 10 mM citrate buffer (pH 6.0) at 60 °C for 5 h for antigen retrieval. After blocking the sections with 1% BSA for 30 min, immunohistochemical staining for sclerostin and β-catenin was performed by incubation with anti-sclerostin (1:100 dilution; R&D Systems; AF1589) and anti-β-catenin (1:100 dilution; ABCAM; ab16051) primary antibodies, respectively, overnight at 4 °C. Detection of the primary antibodies was accomplished using a secondary goat anti-rabbit antibody (Dako) and DAB. Some sections were incubated without the primary antibody and served as negative controls (Online Resource 2). The immunolabeled sections were counterstained with hematoxylin and eosin (H&E) stain and photographed using a ×20 objective with Leica DM RA microscope. The number of sclerostin and β-catenin–positive (brown staining) osteocytes were counted using ImageJ software in a blinded manner. The percentage of positive cells was calculated as the number of positive cells divided by the total number of cells (positive plus negative). The positively stained cells were given a score based on the staining intensity (mild staining, scored 2; strong staining, scored 5). The following formula has been used to generate the staining score:

$${\text{Staining score}}\,=\,({\text{percentage of non-stained cells or empty lacunae}}\, \times \,0)\,+\,({\text{percentage of mildly staining lacunae}}\, \times \,{\text{2}})\,+\,({\text{percentage of strongly staining lacunae}}\, \times \,{\text{5}}).$$

### Statistics

Statistical analyses were performed using GraphPad Prism version 6.0 for Windows (GraphPad Software, La Jolla, CA, USA). *T* tests were used to assess the effect of lactation on the morphometric parameters of osteocyte lacuna network. Differences in gene expression and protein production between loaded and non-loaded fibulae were tested using Student’s two-tailed *t* test. The sclerostin and β-catenin expression data were also presented as relative differences between the loaded and non-loaded fibula to examine whether the response of osteocytes to loading is stronger in the lactating than the virgin mice using Student’s two-tailed *t* test. Normality of the distributions was assessed using a Kolmogorov–Smirnov test. In the case of non-normally distributed data, a nonparametric test was used. All tests were considered significant at two-tailed *p* < 0.05. Data are presented as mean ± SD. Technical difficulties resulted in unequal sample size between the groups (Table 1).

**Table 1:** Sample size

	Virgin		Lactation	
	Loaded	Non-loaded	Loaded	Non-loaded
Sclerostin	8	6	7	4
β-catenin	6	6	4	4

## Results

### Osteocyte Lacunar Volume Increased During Lactation

**Fig. 2:**
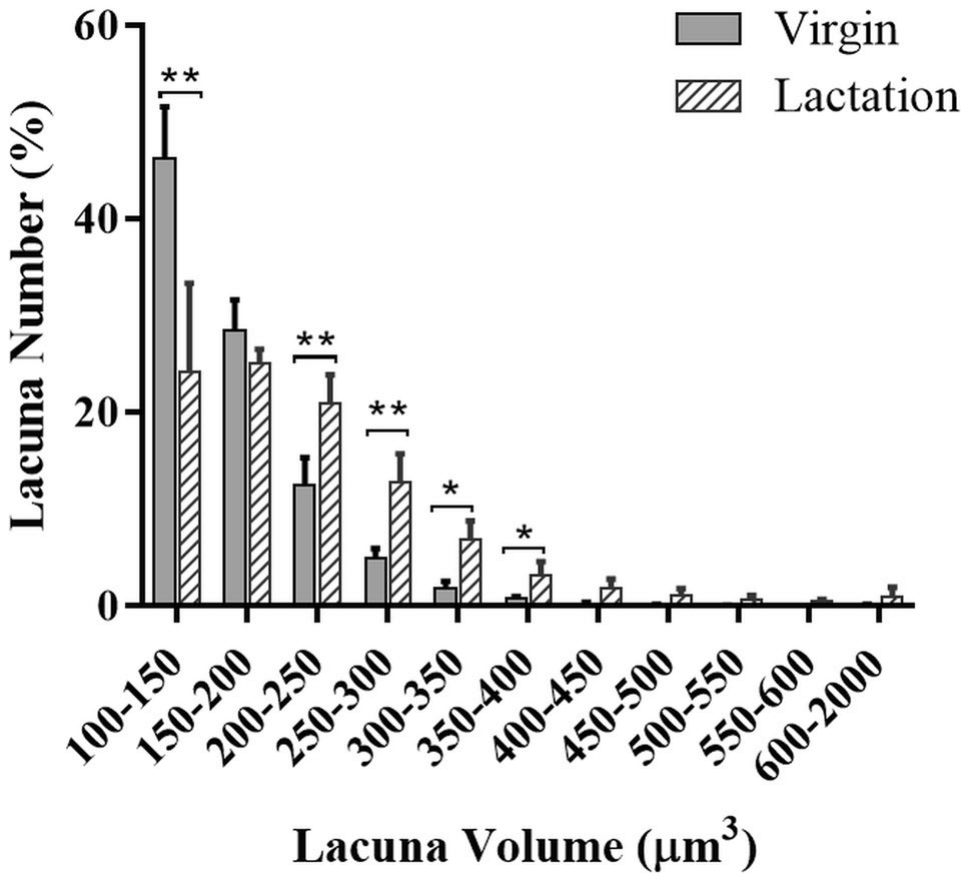
Lacuna volume distribution. Distribution of osteocyte lacuna volume in range between 100 and 2000 µm^3^ indicating significant difference in lacuna volume between virgin and lactating groups. *p < 0.05 and **p < 0.01

To determine whether there are significant differences in osteocyte lacuna shape between virgin and lactating mice, morphometric parameters of osteocyte lacuna network in the fibulae were analyzed using Nano-CT. Lacuna porosity significantly increased during lactation (*p* < 0.01; Table 2). The mean lacuna volume in lactating mice (218.2 ± 27.4 µm^3^) was significantly larger than in virgin mice (163.5 ± 6.3 µm^3^). With lactation, the number of small lacunae (up to 200 µm^3^) reduced, whereas the number of larger lacunae (larger than 200 µm^3^) increased (Fig. 2). The lacunar density (the number of lacunae per bone volume) was unaffected. No significant variations were found in mean lacuna orientation as well as sphericity. Therefore, the fibulae of lactating and virgin mice were selected to investigate the effect of lacuna shape on the responses of osteocytes to the mechanical loading.

**Table 2:** Morphometric parameters of lacunar network

Parameters	Virgin	Lactation	p (*t* test)
Total microporosity (%)	1.33 ± 0.43	1.88 ± 0.68	0.20
Ca.V/ Ct.TV (%)	0.66 ± 0.34	0.79 ± 0.46	0.70
Lc.V/Ct.TV (%)	0.66 ± 0.14	1.09 ± 0.22	0.02
N.Lc/Ct.TV (mm^−3^)	40,387 ± 7448	49,437 ± 4122	0.08
<Lc.V> (µm^3^)	163.5 ± 6.4	218.2 ± 27.4	0.02
<Lc.Sph>	0.71 ± 0.01	0.70 ± 0.03	0.80
<Lc.θ> (°)	11.49 ± 0.90	11.40 ± 0.77	0.90

Total microporosity = (Ca.V + Lc.V)/Ct.TV; Ca.V/Ct.TV = canal volume density; Lc.V/Ct.TV = lacuna volume density; N.Lc/Ct.TV = lacuna number density; <Lc.V > = mean lacuna volume; <Lc.Sph > = mean lacuna sphericity; <Lc.θ> = mean lacuna orientation. Values are mean ± SD

### Mechanical Loading Downregulated *Sost* and Sclerostin Expression and Upregulated β-catenin Expression by Osteocytes

**Fig. 3:**
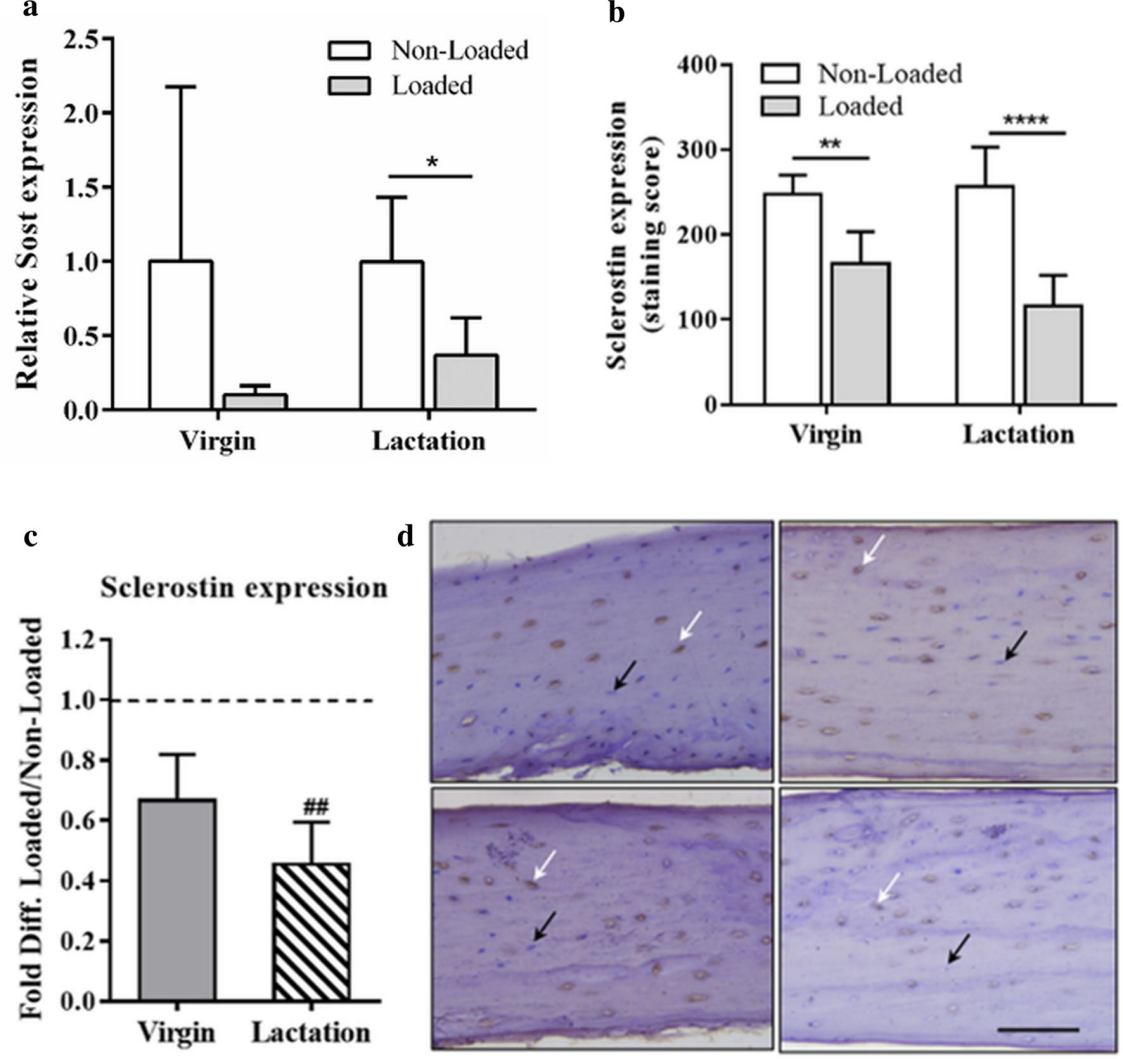
Effect of lacuna morphology (lactate vs. virgin) on the load-induced changes in the *Sost* gene expression and sclerostin expression by osteocytes. **a** Mechanical loading significantly decreased *Sost* by ~ threefold in fibulae of lactating mice. **b** Mechanical stimulation inhibited sclerostin expression in osteocytes in both virgin and lactating mice. **c** Data are expressed as fold decrease compared to non-loaded groups. Lactating mice with enlarged lacuna network showed stronger loading-induced reduction in sclerostin expression in osteocytes. **d** Sclerostin immunohistochemistry. Histological sections from the distal fibula diaphysis of virgin non-loaded (upper left) and loaded (lower left) and lactating non-loaded (upper right) and loaded (lower right) fibulae. The white arrows show sclerostin-positive osteocytes, while the black arrows demonstrate sclerostin-negative osteocytes. The percentage of positive cells was calculated as the number of positive cells divided by the total number of cells (positive plus negative). The positively stained cells were given a score based on the staining intensity (mild staining, scored 2; strong staining, scored 5). Values are mean ± SD. Significant effect of mechanical loading, *< 0.05, **p < 0.01, ****p < 0.0001. Significant effect of lactation with enlarged lacuna network, ^##^*p* < 0.01. Scale bar = 100 µm

**Fig. 4:**
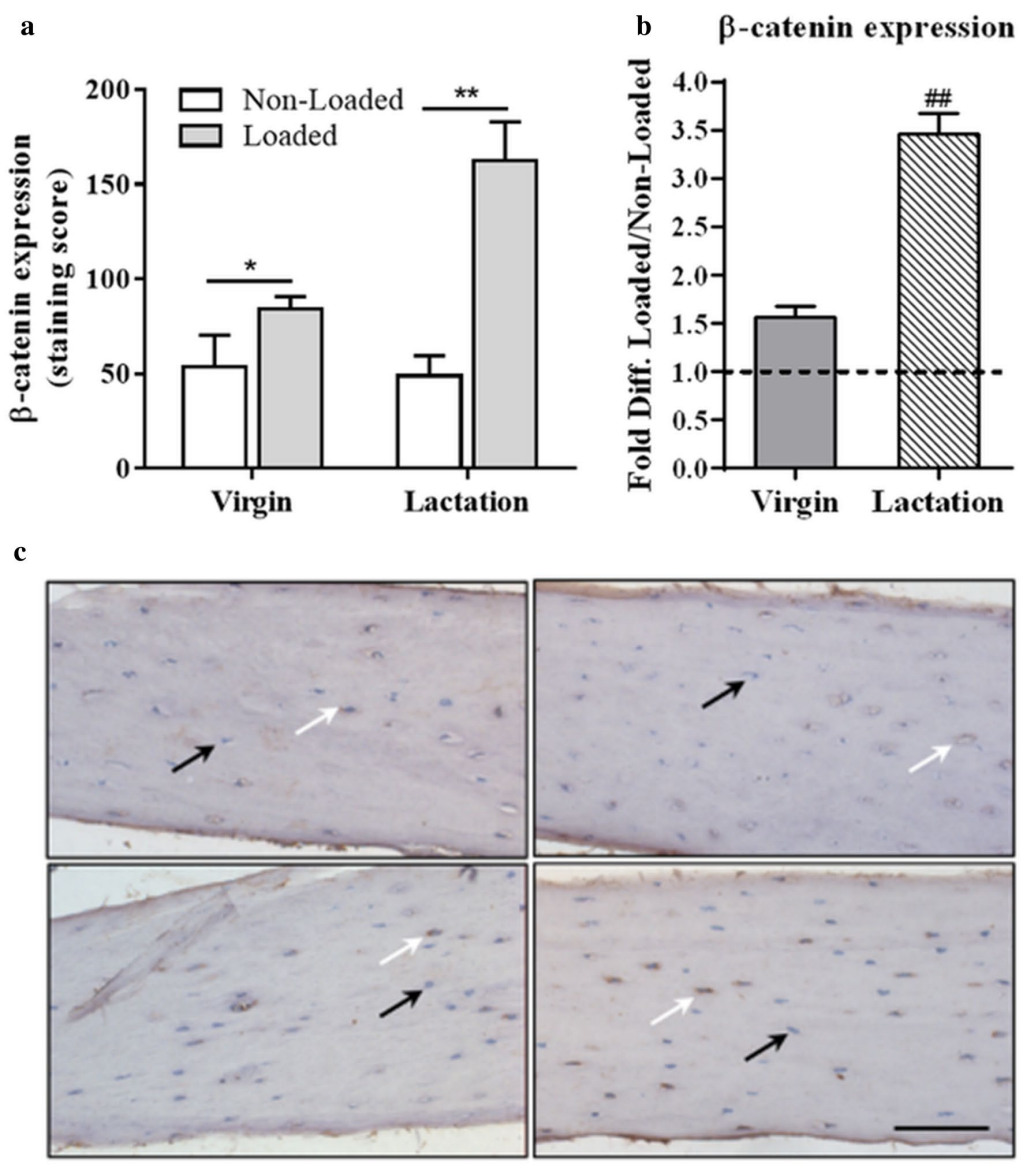
Effect of lacuna morphology (lactate vs virgin) on the load-induced changes in the β-catenin expression by osteocytes. **a** Mechanical stimulation enhanced β-catenin expression in osteocytes in both virgin and lactating mice. **b** Data are expressed as fold increase compared to non-loaded groups. Lactating mice with enlarged lacuna network showed stronger loading-induced increase in β-catenin expression in osteocytes. **c** β-catenin immunohistochemistry. Histological sections from the distal fibula diaphysis of virgin non-loaded (upper left) and loaded (lower left) and lactating non-loaded (upper right) and loaded (lower right) fibulae. The white arrows show β-catenin-positive osteocytes, while the black arrows demonstrate β-catenin-negative osteocytes. The percentage of positive cells was calculated as the number of positive cells divided by the total number of cells (positive plus negative). The positively stained cells were given a score based on the staining intensity (mild staining, scored 2; strong staining, scored 5). Values are mean ± SD. Significant effect of mechanical loading, *p < 0.05, **p < 0.01. Significant effect of lactation with enlarged lacuna network, ^##^*p* < 0.01. Scale bar = 100 µm

Next, we investigated the effect of mechanical stimulation on *Sost* gene expression and the sclerostin and β-catenin amount in osteocytes in situ in fibulae from virgin and lactating mice. Quantitative PCR analysis showed that *Sost* gene expression was significantly decreased ~ threefold by the mechanical loading of fibulae in lactating mice (Fig. 3a). The effect of loading on Sost expression in virgin mice did not reach significance, although a clear trend was visible (Fig. 3a). We found no significant difference in the magnitude of the *Sost* response to mechanical loading between lactating and virgin mice. Immunohistochemical staining revealed that the mechanical loading of fibulae significantly decreased the sclerostin expression in the osteocytes in both virgin (*n* = 7) and lactating mice (*n* = 8) compared to unloaded fibulae (virgin, *n* = 4; lactating, *n* = 6), as shown in Fig. 3b, d. We further found that β-catenin was significantly increased in osteocytes in situ in fibulae from both virgin (*n* = 4) and lactating mice (*n* = 6) during mechanical loading compared to static conditions (virgin, *n* = 4; lactating, *n* = 6), as shown in Fig. 4a, c.

### Osteocyte in Fibulae of Lactating Mice Showed a Stronger Response to Mechanical Loading as Compared to Virgin Mice

To determine whether osteocytes in larger lacunae show stronger response to mechanical loading, relative differences in sclerostin and β-catenin production by osteocytes between loaded and non-loaded fibulae were compared between virgin and lactating mice containing enlarged lacuna network. The loading-induced decrease in sclerostin expression by osteocytes was smaller in virgin mice (33% decrease ± 15, *n* = 7) than lactating mice (55% decrease ± 14, *n* = 8), demonstrating that a stronger loading-induced reduction in sclerostin expression is associated with an enlarged lacuna network (Fig. 3c). In addition, mechanical loading upregulated β-catenin expression in osteocytes in lactating mice by 3.5-fold [± 0.2 (± SD), *n* = 6] which is significantly (*p* < 0.01) higher than the fold increase in β-catenin in osteocytes of fibulae from virgin mice (1.56 ± 0.12, *n* = 4), supporting stronger response of osteocytes with enlarged lacunae to the mechanical loading as compared to osteocyte with smaller lacunae (Fig. 4b).

## Discussion

In this study, we aimed to quantify the response of osteocytes in mechanically loaded fibulae from lactating and virgin mice of similar age, gender, and genetic background, having different lacunar morphology, as measured by quantifying protein expression of the established mechanoresponsive proteins sclerostin and β-catenin as well as *Sost* gene expression. We found a stronger loading-induced reduction in sclerostin and stronger increase in β-catenin expression by osteocytes residing in the fibulae of lactating mice as compared to the response in virgin mice. Mechanical loading significantly affected *Sost* expression in lactating mice not in virgin mice, indicating that osteocytes in fibulae from lactating mice may respond better to mechanical loading. Hence, our experimental findings support the idea that lacunar morphology affects osteocyte signaling in situ.

In order to investigate the effect of lacunar morphology on the osteocyte mechanoresponse, we chose lactating mice wherein the osteocytes enlarge their lacuna volume by removing bone from their perilacunar bone matrix in a process called osteocytic osteolysis, in response to increased demand for calcium during lactation [26, 27]. Utilizing nano-CT to quantify lacunar morphology, the present study further supports previous findings of lactation-induced osteocytic osteolysis [27] and demonstrated significantly larger lacunae in the fibulae of lactating mice as compared to virgin mice.

For this study, we took advantage of murine fibulae as their small size facilitated analysis by Nano-CT since a single field of view encompassed the entire bone’s cross section; hence no cutting nor preparation was required. Furthermore, the fibula is a load-bearing bone that adapts to mechanical loading; indeed, several groups have successfully used murine fibulae to study skeletal mechanobiology and demonstrated bone adaptation to mechanical loading in a similar way as in the tibia [36–39]. In addition, the fibula is small enough to allow osteocytes to survive in their matrix for up to several days in the absence of blood flow. Accessing the viability of human osteocytes in bone chips, similar in size to our mouse fibulae, as quantified by live-dead staining as well as expression of osteocyte-specific markers shows that 60% of the osteocytes are alive and functional after 7 days of culture [40]. This indicates that osteocytes in their native matrix can remain alive up to 7 days, which is much longer than the 48 h after excision of the fibulae when our experiments were terminated.

A limitation of using the lactating mouse model is the obvious hormonal differences between lactating and virgin mice. The most important differences are the higher PTHrP and prolactin levels in lactating mice. In order to diminish the effect of hormones in lactating group, all fibulae were isolated from the body and incubated in culture medium without hormones 24 h before applying ex vivo mechanical loading to equalize conditions before and during the application of the mechanical stimulus. Through this procedure, the direct effect of hormones has been limited, but are unlikely to be completely nullified. There is accumulating evidence that estrogen receptor-α (ERα), which is regulated by estrogen, contributes to the response of bone cells to mechanical strain [4–6]. Pregnancy is associated with high estrogen levels, the major hormonal regulator of bone metabolism [4, 27]. Estrogen levels then rapidly (within a day) return to baseline levels post partum. It has been suggested that dropping estrogen levels cause a change in setpoint of the mechanostat, rendering osteocytes *less* responsive to mechanical stimuli. A drop in estrogen levels would likely decrease osteocyte mechanosensitivity in lactating mice, which is opposite to our findings. Thus, it is unlikely that changes in estrogen levels explain our results. Little to nothing is known regarding the effect of prolactin on the osteocyte mechanoresponse. The PTH type 1 receptor on the other hand has been suggested to be an important component of mechanical signal transduction in osteocytic MLO-Y4 cells [7], but PTHrP and mechanical loading restore bone mass and strength in a diabetic mouse in an additive manner rather than a synergistic manner [41], suggesting that PTHrP does not alter the inherent response of osteocytes to mechanical stimuli. This makes it even less likely that altered PTHrP levels explain our results. Lytic enzymes that are upregulated in osteocytes exposed to hormones during lactation include TRAcP, cathepsin K, and MMP13. MMP14 has been shown to affect the response of osteocytes to mechanical loading in vitro [42], suggesting that certain types of MMPs may affect the osteocyte mechanoresponse. However, in order to affect the mechanoresponse, molecules need to be in a molecular favorable position, such as the transmembrane proteins integrins. MMP14 is such a transmembrane protein, while TRAcP, cathepsin K, and MMP13 are not. Together, these data make it likely that the enhanced response of osteocytes in fibulae of lactating mice is due to the enlarged lacunae rather than the direct effect of altered hormone levels on the osteocyte mechanoresponse. Further studies need to investigate whether expression of genes activated by prolactin and PTHrP persisted in the osteocytes of lactating mice, in order to exclude the possibility that these hormones enhance the mechanical response, separate from changing the lacuna morphology.

Using fibulae has some limitations. The very small size of tiny mouse fibulae leads to difficulties with paraffin sectioning resulting in relatively low number of good sections. Furthermore, the straight alignment of fibula during gluing the sample in the bone holder is important as a skewed position would affect the strain distribution in the fibula during compression loading and therefore the osteocyte response to mechanical loading. This is difficult to achieve with a small and fragile bone such as the fibula. We discarded any samples which were not perfectly aligned, thereby ensuring validity of our experiments. These two technical issues caused the unequal sample sizes between the groups. In addition, in this study, we focused our quantification of lacuna size at the distal part of the fibula and we do not know whether lactation also affects the size of lacunae in the middle and proximal parts of fibulae. Since Sost gene expression was measured in osteocytes residing through the whole fibula, it is possible that a lack of difference in lacuna size in the proximal side of the fibulae between lactating and virgin mice caused the non-significant difference in the magnitude of *Sost* response to mechanical loading between lactating and virgin mice.

Finally, a limitation of this study is that we have used antibodies for visualization of β-catenin that do not discriminate between the phosphorylated and unphosphorylated form. However, it has been well established that mechanical loading in bone leads to inhibition of GSK-3β, via phosphorylation of AKT, initiated by factors such as nitric oxide, focal adhesion kinase, and prostaglandin E_2_ in response to mechanical stimuli [43–46]. As a result of GSK-3β inhibition, β-catenin stays unphosphorylated upon a mechanical stimulus, is no longer targeted for degradation, accumulates inside the mechanically stimulated osteoblast or osteocyte, and finally initiates gene transcription. Increases in β-catenin protein expression in these cases are thus not to be attributed to changes in mRNA expression. Therefore, we chose to measure β-catenin only at the protein level. Production of canonical Wnts by mechanically loaded cells could in theory also contribute to inhibition of GSK-3β in osteocytes, and subsequent accumulation of β-catenin, although a study by Lara-Castillo et al. [43] suggests that Wnts play no role in the increase in β-catenin as observed in osteocytes in vivo in response to loading. Increases in β-catenin levels as observed in response to mechanical stimuli can thus almost certainly be attributed to accumulation of the unphosphorylated (active) form.

Using micro-finite element modeling, we previously demonstrated that the lacunar geometrical features can affect the mechanical environment of the osteocytes, such that lacunae with larger volume experience higher maximum effective strains than lacuna with small volume. Osteocytes exposed to higher maximum effective strains respond stronger than those exposed to lower strains [25], thus at the same overall load placed on bone, bones with larger lacunae are expected to elicit a stronger response in the osteocytes. This is in line with the experimental findings as obtained in the present study.

We previously showed that the osteocyte lacunae are becoming smaller and more spherical with aging [19, 28]. The findings of the present study indicate that bones with smaller lacunae respond less to mechanical loading than bones with larger lacunae, suggesting that smaller lacunae in aged bones are a potential causative factor for the reduced bone mechanoresponsiveness with aging. Our findings may also explain the slower orthodontic tooth movement in elderly as compared to young people. Orthodontic tooth movement is a biological consequence of alveolar bone remodeling induced by mechanical force and controlled by osteocytes [47]. Previous studies have shown that parathyroid hormone treatment [48, 49], lactation, and chronic dietary deficiency of calcium [50] could accelerate the rate of tooth movement through enhancement of alveolar bone remodeling process. We are well aware that all of these interventions potentially directly affect osteocytes, osteoblasts, or osteoclasts, so no strong conclusions with regard to osteocyte mechanosensing are drawn. Yet, all of these factors are also associated with osteocytic osteolysis [26] and enlargement of the lacunar network. Thus, these findings, at the very least, do not oppose our hypothesis that osteocytes residing in enlarged lacunae show a stronger mechanoresponse.

In summary, we found that the osteocytes in fibulae of lactating mice, which are embedded in larger lacunae, show a greater response to mechanical loading in comparison with the osteocytes residing in fibulae of virgin mice. Our results support the theory that osteocyte lacunar morphology affects bone mechanotransduction. Given the ability of osteocytes to orchestrate osteoblast and osteoclast behavior, and their ability to shape their immediate microenvironment, a better understanding of the relationship between osteocyte lacunar shape and bone mechanobiological response may reveal whether altered lacunar shape is one of the pathways involved in lactation, disease, and age-related bone loss.

## Electronic supplementary material

Below is the link to the electronic supplementary material.

Supplementary material 1 (PDF 195 KB)

Supplementary material 2 (PDF 184 KB)
