# Supplementary material for: Mechanical Loading Differentially Affects Osteocytes in Fibulae from Lactating Mice Compared to Osteocytes in Virgin Mice: Possible Role for Lacuna Size
Source: Calcif Tissue Int. 2018 Aug 14;103(6):675–85. doi: 10.1007/s00223-018-0463-8 (PMC6208961; doi:10.1007/s00223-018-0463-8)
Supplement: Supplementary file 1 — Supplementary material 1 (PDF 195 KB) [file 223_2018_463_MOESM1_ESM.pdf]

Article title: MECHANICAL LOADING DIFFERENTIALLY AFFECTS OSTEOCYTES IN FIBULAE FROM LACTATING MICE COMPARED TO OSTEOCYTES IN VIRGIN MICE: POSSIBLE ROLE FOR LACUNA SIZE

Journal name: Calcified Tissue International

Author names: Haniyeh Hemmatian[1,2], Rozita Jalali[2], Cor M. Semeins[2], Jolanda M.A. Hogervorst[2], G. Harry van Lenthe[1], Jenneke Klein-Nulend[2], Astrid D. Bakker[2]

Affiliation:

[1] Biomechanics Section, Department of Mechanical Engineering, KU Leuven, Leuven, Belgium

[2] Department of Oral Cell Biology, Academic Centre for Dentistry Amsterdam (ACTA), University of Amsterdam and Vrije Universiteit Amsterdam, Amsterdam Movement Sciences, Amsterdam, The Netherlands

Corresponding author:

Jenneke Klein-Nulend

Email: j.kleinnulend@acta.nl

Supplementary data

Validation study 1

Male six-weeks-old C57BL/6 mice (VU University Amsterdam, Amsterdam, The Netherlands) were used. Ethical approval for the animal procedures was given by the Animal Ethics Committee of VU University Amsterdam (ACTA-2014-01). Animals were kept in accordance with the regulations of the Animal Welfare committee of the VU University Amsterdam (ACTA14-02). Mechanical stimulation was performed for 5 minutes at 5 Hz with a calculated displacement to achieve 3000 με strain. Total RNA was isolated 4 hours after mechanical stimulation of the ulnae. Loaded and non-loaded ulnae were crushed with a small pestle after placement in a 1.5 ml eppendorf tube containing Qiagen lysis buffer (Qiagen, Gaithersbrug, MD, USA) and 2 β-mercaptoethanol. RNA isolation was performed according to the Qiagen protocol with the RNeasy® Mini Kit (Qiagen,Gaithersbrug, MD, USA). Isolated samples were checked for purity by calculation of the A260/280 ratio. To remove any genomic DNA, samples were treated with DNase according to the Qiagen DNase protocol. Synthesis of cDNA was performed by using the first strand cDNA synthesis kit (Fermentas, Thermo Fisher scientific®, Waltham, MA, USA). The reverse transcriptase mix contained 9 μl RNA, 1 μl oligo(dT18), 1 μl D(N)6, 4 μl buffer, 2 μl dNTPs, and 1 μl RNAse Inhibitor. The PCR reaction was performed with the Lightcycler 480® RT-Kit. One reaction for a 384 wells plate contained 5 μl Lightcycler® 480 SYBR master 1, 0.375 μl forward primer, 0.375 μl reverse primer, 3.25 μl distilled water, and 1 μl cDNA. Primer sequences were as follows: Wnt 3a, forward: 5’-CCATGAACCGTCACAACAA-3’, reverse: 5’-TGTTTCTCTACCACCATCT-3’; Wnt 3a (multiple species), forward: 5’-CCATGAACCGCCACAACAA-3’, reverse: 5’-TGTTTCTCCACCACCATCT-3’; Wnt 5a, forward: 5’-GTGGTCTCTAGGTATGAATAA-3’, reverse: 5’-

CGCGTACGTGAAGGCCGTC-3’; DMP1, forward: 5’-CGGCTGGTGGACTCTCTAAG-3’, reverse: 5’-CGGGGTCGTCGCTCTGCATC-3’; RUNX2, forward: 5’-ATGCTTCATTCGCCTCAC-3’, reverse: 5’-ACTGCTTGCAGCCTTAAAT-3’; MEPE, forward: 5’-GGAGCACTCACTACCTGAC-3’, reverse: 5’-TAGGCACTGCCACCATGT-3’; PBGD, forward: 5’-AGTGATGAAGATGGACAACT-3’, reverse: 5’-TCTGGACCATCTTCTTGCTGA-3’; M2B, forward: 5’-TGCTATCCAGAAACCCCTCAA-3’, reverse: 5’-GCGGGTGGAACTGTGTTACG-3’; YWHAZ, forward: 5’-GATGAAGCCATTGCTGAACTTG-3’, reverse: 5’-CTATTTGTGGGACAGCATGGA-3’. One reaction for a 96 wells plate contained 10 μl Lightcycler® 480 SYBR master 1, 1 μl forward primer, 1 μl reverse primer, 6 μl distilled water, and 2 μl cDNA. Analysis of relative gene expression was performed with Lightcycler software. A baseline was set after all the samples were selected, and concentrations were calculated with the fit point method with an imported standard curve. PCR product amplification was checked by running the samples on a 1% agarose gel. gene expression was analyzed with the Wilcoxon matched-pairs signed rank test. This resulted in a significant difference ($p<0.05$) between Wnt-3a gene expression in non-loaded and loaded bones (Fig. S1).

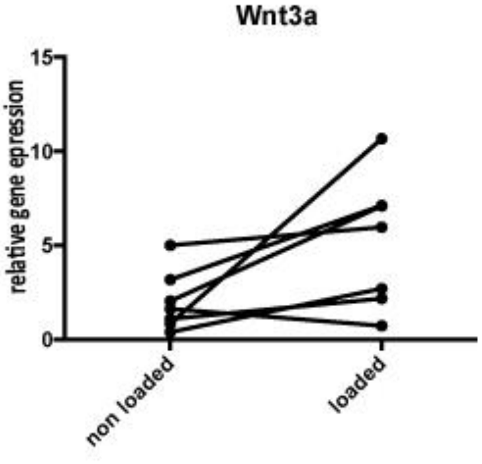

Fig S1. Relative Wnt3a gene expression under loaded and non-loaded conditions. Gene expression is normalized against YWHAZ and BPGD. The lines between non-loaded and loaded data connect data obtained with fibulae from one mouse.

Validation study 2

Fibulae were dissected from 2 month old adult male C57bl/6 mice. Each fibula was glued in a bone holder with cyanoacrylate instant adhesive (Permabond, St. Pottstown, PA, USA). The samples were then incubated in Phenol-red free DMEM (Gibco, Paisley, UK) supplemented with 100 U/mL penicillin (Sigma), 0.05 mg/ml streptomycin sulphate (Sigma), 1.25 µg/ml fungizone (Gibco), 5 µg/ml Vitamin C (Sigma) and 0.2% Bovine Serum Albumin (BSA) for 23h prior to mechanical loading. For DAR-4M AM chromophore loading, fibulae were incubated in 10 µM DAR-4M AM dye for 1 h at room temperature in DMEM without phenol red. Fibulae were gently washed with D-MEM without phenol red, and were mechanically stimulated as follows: sinusoidal displacement of the fibulae varying from 0–12.5 µm, for 5 min at 5 Hz. This displacements corresponds to a theoretical bulk mechanical load of 3000µε Nitric oxide (NO) production was monitored by taking images of the same area of the bone before loading, directly after loading, and after saturation of the NO signal with external NO released from the NO donor S-nitroso-N-acetyl-penacillamine (SNAP 10 mM; Sigma, St. Louis, MO, USA). Images were taken using a BioRad MRC-1000 UV Leica confocal system attached to a Leica inverted microscope (Leica Microsystems, Wetzlar, Germany). A Leica 20x objective lens was used. The excitation wavelength used was 545 nm, and emission spectra peaked at 580 nm. The NO production before and directly after mechanical stimulation of individual osteocytes in situ was semi-quantified as percentage of the maximum fluorescence intensity in that cell after saturation using Leica Confocal Software (Leica Microsystems, Wetzlar, Germany).

Global matrix strains were measured using EA-06-015LA-120 strain gauges (Vishay Micro-Measurements, Raleigh, North Carolina, USA) in a quarter bridge configuration containing one active gauge element, and temperature compensation. Strain gauges were glued to the approximate mid-shaft of the fibula. Calculations were done using the recordings

made between 9 and 11 seconds after start of mechanical stimulation (2 seconds, 10 cycles). Strains were calculated using a simplified formula, since the contribution of temperature changes to the outcome of our measurement was negligible. We simplified the formula to:

$$\frac{V_o}{V_{ex}} = \frac{GF \times \varepsilon}{4}$$

Where ε equals the strain (dimensionless) and GF the gauge factor (2.06). V_0 and V_{ex} are given in Volt.

Result: The applied load resulted in corresponding strains of maximum $3x10^3$ ($\pm 0.7x10^3$) με. Considering that the fibulae were cut to an approximate length of 5 micrometer, this is in the same ballpark as expected. Mechanical stimulation resulted in a 1.3-fold increase in DAR fluorescence intensity by osteocytes compared with its non-stimulated state indicating a rapid increase in NO production by osteocytes in response to mechanical loading in situ.

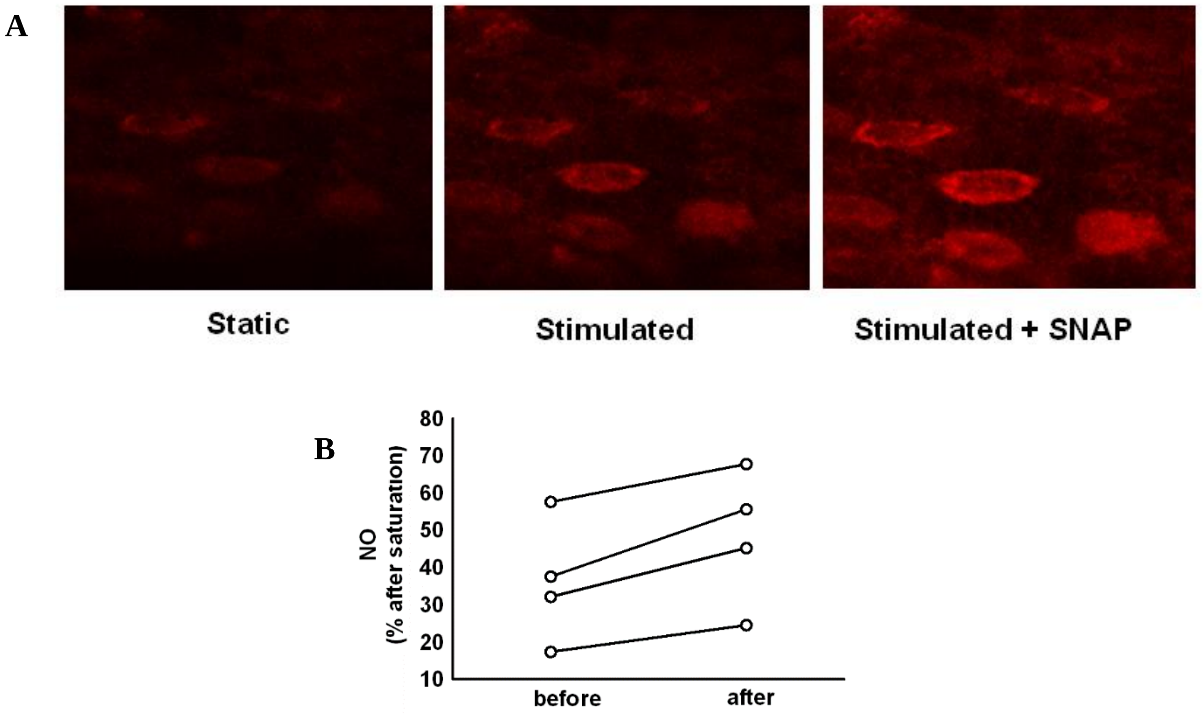

Fig S2. Effect of mechanical stimulation of fibulae on NO production in osteocytes in situ. A) Representative fluorescence images of DAR-4M AM chromophore loaded osteocytes in

situ shows upregulation of DAR fluorescence intensity after mechanical stimulation of fibula ex vivo. B) Relative fluorescent intensity, indicating NO production, increased after a displacement of 12.5 µm. Original magnification, x20.
