# Supplementary material for: Mechanical Loading Differentially Affects Osteocytes in Fibulae from Lactating Mice Compared to Osteocytes in Virgin Mice: Possible Role for Lacuna Size
Source: Calcif Tissue Int. 2018 Aug 14;103(6):675–85. doi: 10.1007/s00223-018-0463-8 (PMC6208961; doi:10.1007/s00223-018-0463-8)
Supplement: Supplementary file 2 — Supplementary material 2 (PDF 184 KB) [file 223_2018_463_MOESM2_ESM.pdf]

Article title: MECHANICAL LOADING DIFFERENTIALLY AFFECTS OSTEOCYTES IN FIBULAE FROM LACTATING MICE COMPARED TO OSTEOCYTES IN VIRGIN MICE: POSSIBLE ROLE FOR LACUNA SIZE

Journal name: Calcified Tissue International

Author names: Haniyeh Hemmatian[1,2], Rozita Jalali[2], Cor M. Semeins[2], Jolanda M.A. Hogervorst[2], G. Harry van Lenthe[1], Jenneke Klein-Nulend[2], Astrid D. Bakker[2]

Affiliation:

[1] Biomechanics Section, Department of Mechanical Engineering, KU Leuven, Leuven, Belgium

[2] Department of Oral Cell Biology, Academic Centre for Dentistry Amsterdam (ACTA), University of Amsterdam and Vrije Universiteit Amsterdam, Amsterdam Movement Sciences, Amsterdam, The Netherlands

Corresponding author:

Jenneke Klein-Nulend

Email: j.kleinnulend@acta.nl

Supplementary data

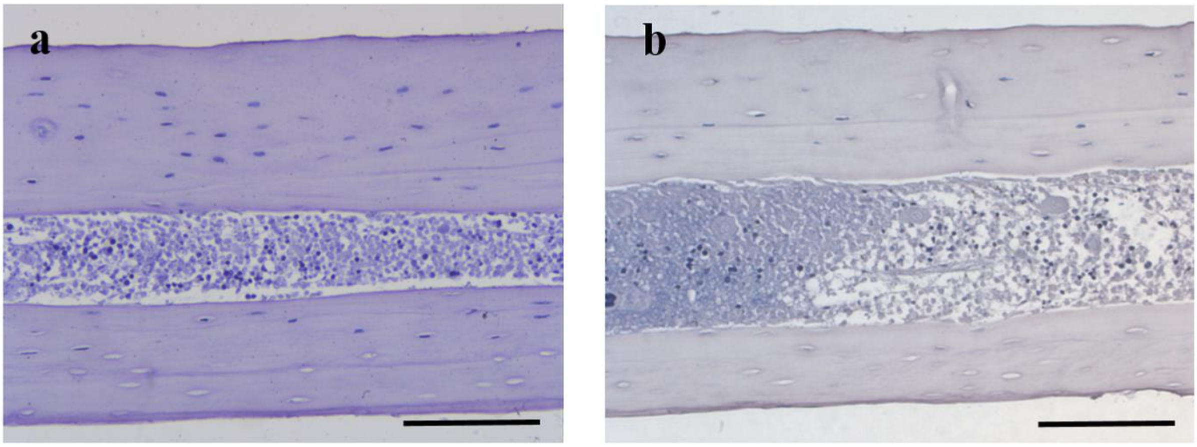

Fig S1. Negative controls for immunohistochemical staining for sclerostin (a) and β-catenin (b).
